# ﻿A survey of the spider genus *Lipocrea* Thorell, 1878 (Araneae, Araneidae) from Guiyang City, Southwest China: An integrated morphological and molecular approach

**DOI:** 10.3897/zookeys.1255.158340

**Published:** 2025-10-10

**Authors:** Jianshuang Zhang, Chengwen Zhang, Yuanqian Xing, Hao Yu, Xiaoqi Mi

**Affiliations:** 1 The State Key Laboratory of Southwest Karst Mountain Biodiversity Conservation of Forestry Administration, School of Life Sciences, Guizhou Normal University, Guiyang, Guizhou, China Guizhou Normal University Guiyang China; 2 College of Agriculture and Forestry Engineering and Planning, Guizhou Provincial Key Laboratory of Biodiversity Conservation and Utilization in the Fanjing Mountain Region, Tongren University, Tongren, Guizhou, China Tongren University Tongren China

**Keywords:** Biodiversity, DNA barcoding, molecular species delimitation, morphology, new species, orb-weaver

## Abstract

A survey was undertaken to study the spider genus *Lipocrea* Thorell, 1878, from Guiyang City, Guizhou Province, southwest China. A total of two species is here addressed based on morphology and five methods of molecular species delimitation, comprising *L. guiyang* J. Zhang, Yu & Mi, **sp. nov.** and *L. fusiformis* (Thorell, 1877), the type species of the genus as well as a new record for mainland China. These two species are distributed in Huaxi District and Kaiyang County of Guiyang, respectively, providing the first formal record of this genus from mainland China.

## ﻿Introduction

*Lipocrea* Thorell, 1878 is a small spider genus that was originally established to include three species: *L. phthisica* (L. Koch, 1871) and *L. tabida* (L. Koch, 1872) from the Australasian Region, and the type species *L. fusiformis* (Thorell, 1877) from the Oriental Region. An additional Oriental species, *L. diluta* Thorell, 1887 was subsequently described. However, the validity of *Lipocrea* as a distinct genus was not recognized by Simon (1887: 187), who treated these species under the genus *Larinia* Simon, 1874. Of the aforementioned species, *L. phthisica* has since been transferred to *Larinia* sensu stricto (Grasshoff 1970). The remaining species, together with *Larinia longissima* (Simon, 1881) from the Ethiopian Region, were subsequently removed from *Larinia* and placed in a newly erected genus, *Larinopa*Grasshoff (1970), by Grasshoff (1970: 226). Although the name *Lipocrea* remains available under the rules of zoological nomenclature, it has been largely overlooked (Levy 1986). The genus *Lipocrea*, as redefined by Grasshoff under the name *Larinopa*, is primarily distinguished from *Larinia* by the structure of the genitalia, especially the morphology of the male palpal organ.

Currently, the WSC (2025) lists five species under *Lipocrea*, none of which are known from the Chinese mainland (WSC 2025). However, in several publications, some of these species are considered to belong to *Larinia*. For example, *L. phosop* (Tanikawa, Into & Petcharad, 2023) was originally described as *Larinia* has not yet been formally transferred to *Lipocrea*. Nevertheless, due to its strong morphological similarity to the type species of *Lipocrea*, *L. phosop* has been provisionally placed in *Lipocrea* by the WSC (2025). Even the type species, *L. fusiformis*, has frequently been placed in *Larinia* and redescribed as such by various authors, including Tanikawa (1989, 2007, 2009), Okuma et al. (1993), Barrion and Litsinger (1995), and Chang and Tso (2004). In view of the above-mentioned, the validity of the genus *Lipocrea* and the current generic placement of its constituent species remains in dispute.

A preliminary genus-level taxonomic molecular analysis of Grasshoff’s (1970) ‘*Larinia* group’ was carried out based on all available COI sequences of the *Larinia* group and related genera (69 from NCBI and 9 newly sequenced here) (Suppl. material 1: table S1). According to the results (Suppl. material 1: fig. S1): (1) the monophyly of the genus *Lipocrea* is well supported; (2) *Larinia* is polyphyletic, which is consistent with the results of Scharff et al. (2020). Based on this preliminary result and considering that a formal comprehensive revision of the *Larinia* group is lacking, we agree with Framenau and Castanheira (2022) that the *Larinia* group requires further systematic study that includes *Larinia lineata* Lucas, 1846 (the type species of the genus). A review of the *Larinia* group is not within the scope of this work. Consequently, the present study assigns the two species treated here to the monophyletic genus *Lipocrea*, rather than to the polyphyletic *Larinia* sensu lato.

**Figure 1.**
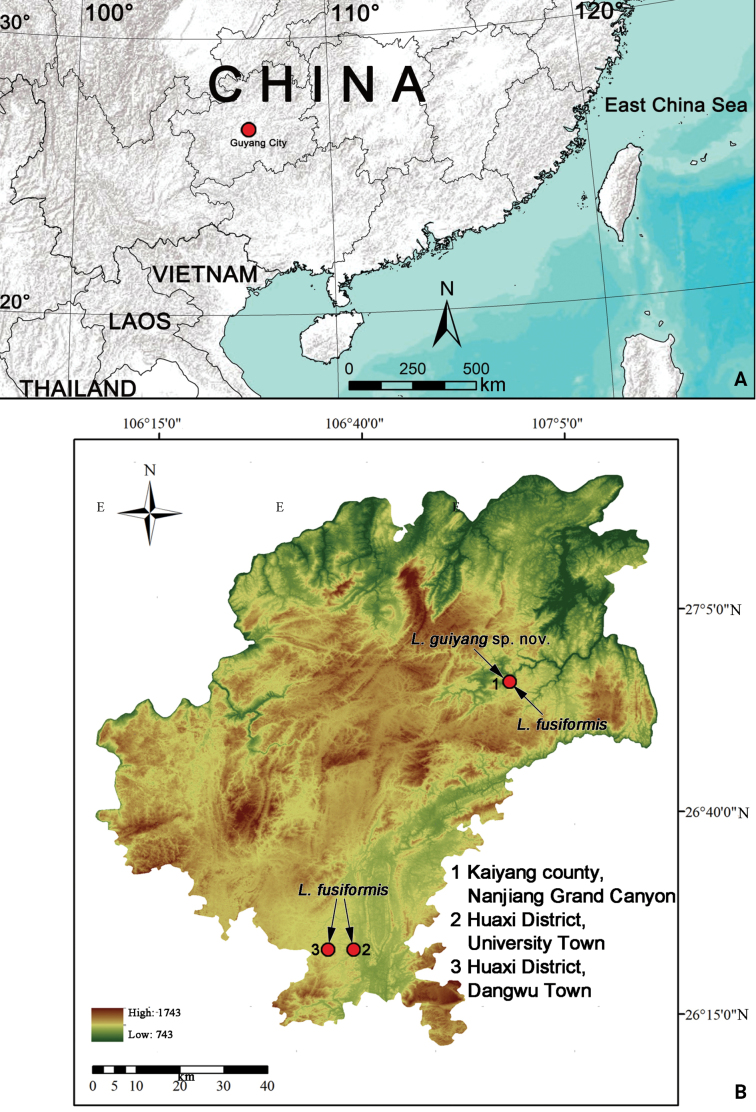
Locality of Guiyang City (A) and distribution records of *Lipocrea* species in Guiyang (B).

Guiyang, the provincial capital of Guizhou Province (Fig. 1A), is the first city to receive the accolade of ‘the national forest city’ in China, famous for more than 55% forest coverage (Zhong 2018). Guiyang is also known as the ‘city of a thousand gardens’, with 1025 urban parks, and is considered one of China’s most biodiverse provincial capitals (Yang 2020; Cheng and Chen 2022). However, spiders can be regarded as being poorly represented in Guiyang, with only 64 species from 25 families recorded or described to date (JZ and HY, unpubl. data). Of these, 21 species are endemic, 16 were newly described, and 15 were reported as new records in recent years (Yu et al. 2018; Yu and Zhang 2019; Xin et al. 2020; Yan et al. 2021; Long et al. 2022; Zhang et al. 2022a, b, 2023; He et al. 2023; Li et al. 2023; Yang et al. 2023a, b, c; Ding et al. 2024; Pan et al. 2024; Jiang et al. 2025; Zhang et al. 2025). This estimate of spider diversity is assumed to be far from the true diversity within this city.

**Figure 2.**
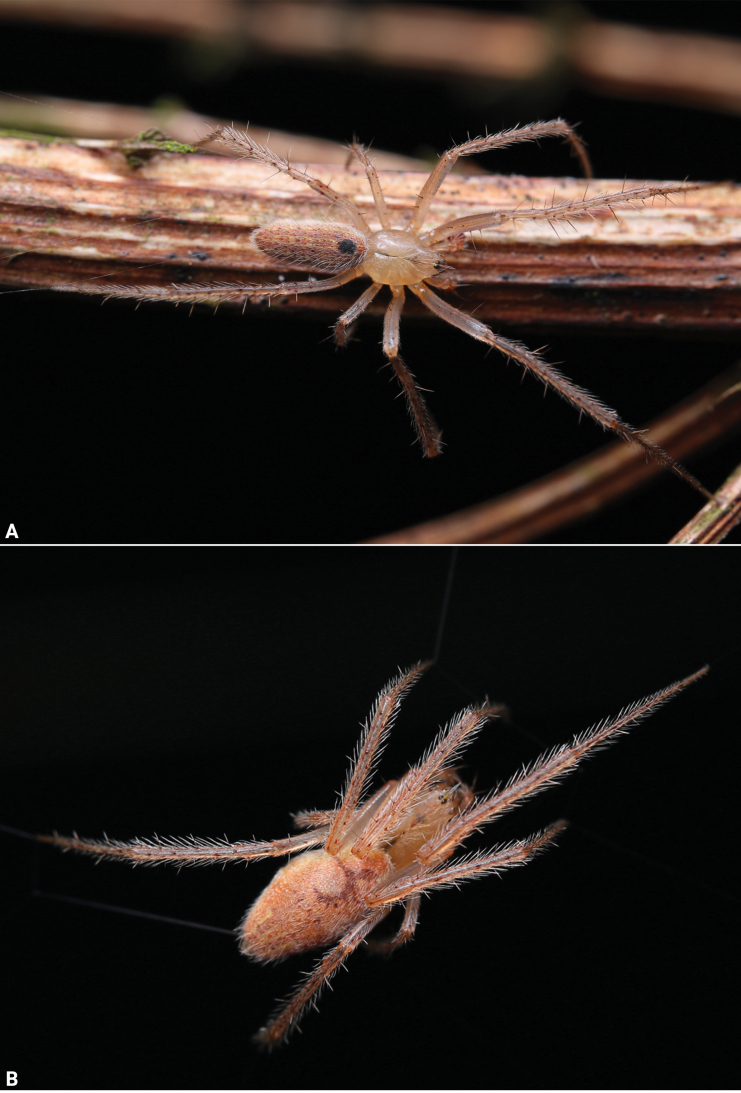
Living specimens of *Lipocrea guiyang* J. Zhang, Yu & Mi, sp. nov. A. Male; B. Female. Photographs by Q Lu (Shenzhen).

**Figure 3.**
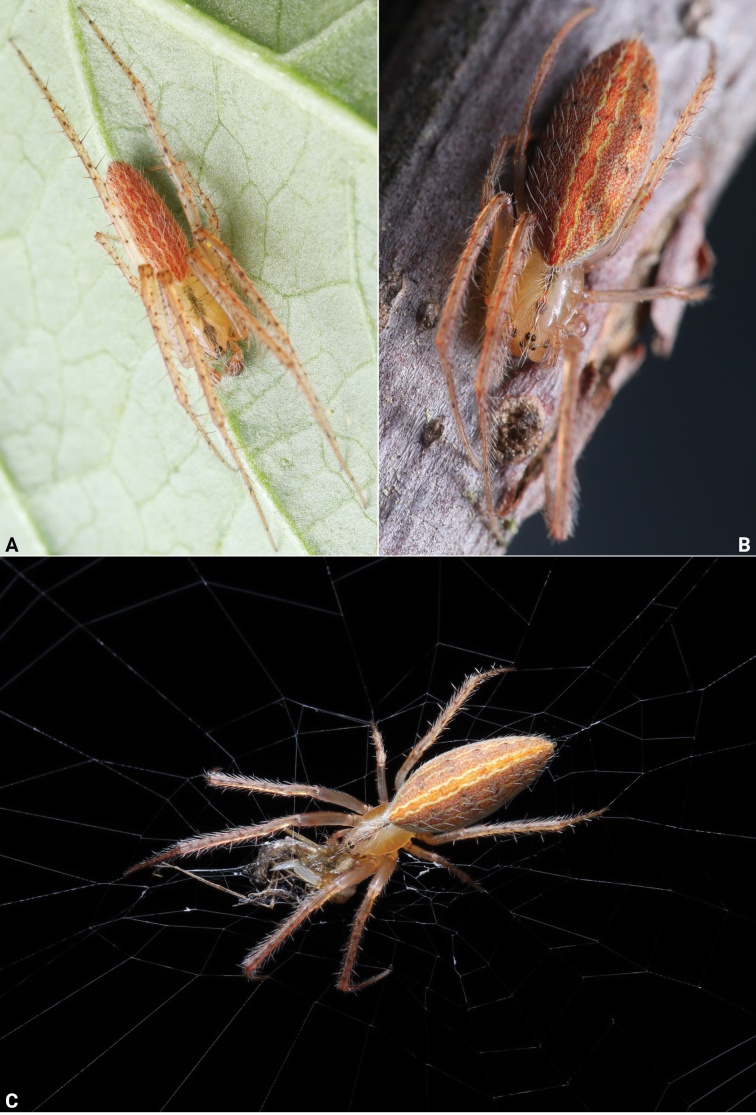
Living specimens of *Lipocrea fusiformis* (Thorell, 1877). A. Male; B, C. Female. Photographs by Q Lu (Shenzhen).

As mentioned above, spiders are poorly studied in Guiyang, including Araneidae. Only five species have been recorded: *Cyrtarachne bufo* (Bösenberg & Strand, 1906), *Hypsosinga pygmaea* (Sundevall, 1831), *Neoscona xishanensis* Yin, Wang, Xie & Peng, 1990, *Nephila pilipes* (Fabricius, 1793) and *Pronoides brunneus* Schenkel, 1936. Recently, short but intensive field collections in Guiyang have been conducted by the staff of the Guizhou Normal University. During these surveys, we have found some *Lipocrea* specimens that belong to at least two morphospecies (Figs 1–3): one is new to science, and the other one has been identified as *L. fusiformis*, a new record for mainland China. The sympatric distribution with *L. fusiformis* and the high intraspecific morphological variation in females of the new species pose significant challenges for sexual pairing and species identification. We therefore generated DNA barcode data to devise a specimen phylogeny and used five molecular species delimitation methods to test morphology-based species identification.

The goal of this paper is to: 1) use the consensus results of an integrated morphological and molecular approach to delimit *Lipocrea* spiders from Guiyang; 2) describe the new species under the name of *Lipocrea guiyang* J. Zhang, Yu & Mi, sp. nov.; 3) re-illustrate *L. fusiformis* based on new material from Guiyang, including supplementary micrographs; and 4) provide a distribution map of *Lipocrea* species in Guiyang City.

## ﻿Materials and methods

### ﻿Taxon sampling

Specimens in this study were collected by hand and by beating vegetation, and directly fixed in absolute ethanol. The right legs were removed and stored at −80 °C for subsequent DNA extraction. The remainder of the specimens was preserved in 80% ethanol for identification and morphological examination. A total of 11 adults were obtained, examined, and processed for DNA extraction but only nine individuals yielded useable DNA (Table 1). All voucher specimens (including types of the new species) are deposited in the Museum of Guizhou Normal University, Guiyang, China (**MGNU**).

**Table 1.** Samples used in species delimitation: specimen label, taxon name, sample collection locality with coordinates, and GenBank accession numbers.

Specimen code	Genus/Species	Sex	Locality	Country	Coordinates	Elevation (m a.s.l.)	COI GenBank accession
YHGY208	*Lipocrea guiyang* sp. nov.	♂	Nanjiang Grand Canyon, Kaiyang County, Guiyang City, Guizhou Prov.	China	26.94°N, 106.97°E	861	PX230067
YHGY209	*Lipocrea guiyang* sp. nov.	♀	Nanjiang Grand Canyon, Kaiyang County, Guiyang City, Guizhou Prov.	China	26.94°N, 106.97°E	861	PX230068
YHGY428	*Lipocrea guiyang* sp. nov.	♀	Nanjiang Grand Canyon, Kaiyang County, Guiyang City, Guizhou Prov.	China	26.94°N, 106.97°E	861	PX230069
YHGY431	*Lipocrea fusiformis*	♂	Dangwu Town, Huaxi District, Guiyang City, Guizhou Prov.	China	26.38°N, 106.60°E	1138	PX230061
YHGY432	*Lipocrea fusiformis*	♀	University Town, Huaxi District, Guiyang City, Guizhou Prov.	China	26.38°N, 106.65°E	1173	PX230062
YHGY433	*Lipocrea fusiformis*	♀	Dangwu Town, Huaxi District, Guiyang City, Guizhou Prov.	China	26.38°N, 106.60°E	1138	PX230063
YHGY495	*Lipocrea fusiformis*	♂	University Town, Huaxi District, Guiyang City, Guizhou Prov.	China	26.38°N, 106.65°E	1173	PX230065
YHGY507	*Lipocrea fusiformis*	♀	University Town, Huaxi District, Guiyang City, Guizhou Prov.	China	26.38°N, 106.65°E	1173	PX230066
YHGY508	*Lipocrea fusiformis*	♀	University Town, Huaxi District, Guiyang City, Guizhou Prov.	China	26.38°N, 106.65°E	1173	PX230064
AT5343	*Lipocrea phosop*	♂	Mai Khet, Mueang Prachinburi District, Prachinburi Prov.	Thailand	14.10°N, 101.33°E		LC756453
AT5337	*Lipocrea phosop*	♀	Mai Khet, Mueang Prachinburi District, Prachinburi Prov.	Thailand	14.10°N, 101.33°E		LC756454
AT5338	*Lipocrea phosop*	♀	Mai Khet, Mueang Prachinburi District, Prachinburi Prov.	Thailand	14.10°N, 101.33°E		LC756455
AT5339	*Lipocrea phosop*	♀	Mai Khet, Mueang Prachinburi District, Prachinburi Prov.	Thailand	14.10°N, 101.33°E		LC756456
AT5340	*Lipocrea phosop*	♀	Mai Khet, Mueang Prachinburi District, Prachinburi Prov.	Thailand	14.10°N, 101.33°E		LC756457
AT5341	*Lipocrea phosop*	♀	Mai Khet, Mueang Prachinburi District, Prachinburi Prov.	Thailand	14.10°N, 101.33°E		LC756458
AT5342	*Lipocrea phosop*	♂	Mai Khet, Mueang Prachinburi District, Prachinburi Prov.	Thailand	14.10°N, 101.33°E		LC756459
AT5345	*Lipocrea phosop*	♂	Ban Pathum, Sam Khok District, Pathum Thani Prov.	Thailand	14.08°N, 100.58°E		LC756460
LJO01	*Larinia joei*	♂	Khlong Sam, Pathum Thani Prov.	Thailand	14.16°N, 100.66°E		LC597525
LJO02	*Larinia joei*	♀	Khlong Sam, Pathum Thani Prov.	Thailand	14.16°N, 100.66°E		LC597526

### ﻿Molecular protocols

Total genomic DNA was extracted using the Cell & Tissue Genomic DNA Isolation Kit (Bioteke, Beijing, China) following the manufacturer’s protocols. We amplified cytochrome c oxidase subunit I (COI) using the primer pairs LCO1490/HCO2198 (Folmer et al. 1994) and standard polymerase chain reaction (PCR) settings (Wheeler et al. 2016). PCR products were transported to the Beijing Tsingke Biotech Co., Ltd. (Chongqing, China) for sequencing using the same PCR primers. We manually edited the sequences using Geneious Prime 2024 (Kearse et al. 2012), translated nucleotide sequences into amino acids to check for stop codons, and ensured the proper configuration of codon positions.

### ﻿Phylogenetic analyses

For phylogenetic analyses, the COI data of 17 specimens of *Lipocrea* were used as the in-group, including eight sequences of *L. phosop* downloaded from GenBank (Tanikawa et al. 2023; NCBI 2025). To root the tree, we included two specimens of *Larinia joei* Tanikawa & Petcharad, 2021 from NCBI (2025) as the outgroup (Table 1). We performed maximum-likelihood (ML) analyses using IQ-TREE v. 2.3.1 (Minh et al. 2020) based on the best COI substitution model (GTR+I+G) in jMODELTEST v. 2.1.10 (Darriba et al. 2012). Branch support was estimated with ultrafast bootstrapping with 1000 replicates (Hoang et al. 2018). Bayesian-inference (BI) was performed with MrBayes v. 3.2.1 (Ronquist et al. 2012) using one independent chain for 50 million generations. The first 10% of trees from each run were discarded as burn-in. Finally, we used FigTree v. 1.4.4 (Rambaut 2012) to visualize and manipulate trees and used Photoshop CC 2018 to summarize them.

### ﻿Molecular species delimitation

To delimit three morphospecies of *Lipocrea* based on an accompanying morphological study of the genus, we used two genetic distance-based methods: the DNA barcoding gap (Barrett and Hebert 2005) and ABGD (Puillandre et al. 2012), as well as three methods based on the inferred tree, GMYC (Pons et al. 2006), P ID (Liberal), and mPTP (Kapli et al. 2017).

Because the P ID (Liberal) and DNA barcoding gap (Barrett and Hebert 2005) methods require a priori designation, we assigned 17 *Lipocrea* individuals to three putative species based on a combination of phylogenetic topology and morphological characteristics. With the DNA barcoding gap, we used the overlap between the interspecies and intraspecies Kimura two-parameter (K2P) and uncorrected *p*-distance for each candidate species calculated in MEGA X (Kumar et al. 2018). The P ID (Liberal) method tests species delimitation by relying on defining the putative species groups. We used the BI tree as a guide to test species hypothesis (Xu et al. 2017).

The other three methods that we used do not require terminals to be a priori assigned to putative species. ABGD calculates all pairwise distances in the data set, evaluates intraspecific divergences, and then sorts the terminals into candidate species with calculated P values. We performed the ABGD analysis on a web server (https://bioinfo.mnhn.fr/abi/public/abgd/) using three different models: Jukes-Cantor (JC69; Jukes and Cantor 1969), K2P (Kimura 1980), and simple distance (*p*-distance; Nei and Kumar 2000). We analyzed the data using two different values for the parameters *P*_min_ (0.001 and 0.0001), *P*_max_ (0.1 and 0.2), and relative gap width (X = 1.5 or 2), with the other parameters set to default values. We used the BI tree as a guide to test the species hypothesis (Xu et al. 2017). Two runs of 100 million steps were used for the mPTP logging every 1 million steps, discarding the first 2 million steps. Each run was started from a random delimitation. The GMYC methodology (Pons et al. 2006) analysis was conducted using the single-threshold model in the “splits” package (Ezard et al. 2009) for R 4.2.2 (R Development Core Team 2024). BEAST 2.6.7 (Bouckaert et al. 2014) was used to produce an ultrametric tree for the GMYC analysis. Analyses were run for 50 million steps with 10% of the trees in each chain discarded as burn-in.

### ﻿Morphological protocols

Specimens were examined using an Olympus SZX7 stereomicroscope. Further details were studied under a CX41 compound microscope. Male and female copulatory organs were examined and illustrated after dissection. Epigynes were removed and cleared in lactic acid or a warm 10% potassium hydroxide (KOH) solution. Images were captured with a Canon EOS 70D digital camera (20.2 megapixels) mounted on an Olympus CX41 compound microscope and assembled using Helicon Focus v. 6.80 image-stacking software. All measurements were obtained using an Olympus SZX7 stereomicroscope and are given in millimeters. Eye diameters were measured at the widest part. The total body length does not include the chelicerae or spinnerets. Leg lengths are given as total length (femur, patella+tibia, metatarsus, tarsus). The terminology used in the text and figure legends follows Grasshoff (1970), Framenau and Scharff (2008), Framenau and Castanheira (2022), Tanikawa and Petcharad (2021), and Tanikawa et al. (2023).

References to figures in the cited papers are listed in lowercase (fig. or figs); figures from this paper are noted with an initial capital (Fig. or Figs). The abbreviations used in the text are:

**AER** anterior eye row;

**ALE** anterior lateral eyes;

**AME** anterior median eyes;

**C** conductor;

**CD** copulatory duct;

**CO** copulatory opening;

**Cy** cymbium;

**Em** embolus;

**FD** fertilisation duct;

**HP** hook-shaped process of MA;

**KP** knob-like projection;

**MA** median apophysis;

**MS** median septum;

**MOQ** median ocular quadrangle;

**Pc** paracymbium;

**PLE** posterior lateral eyes;

**PME** posterior median eyes;

**R** radix;

**RA** radix apophysis;

**RER** posterior eye row;

**Sc** scape;

**Sp** spermatheca;

**St** subtegulum;

**T** tegulum;

**TA** terminal apophysis;

**TA I** terminal apophysis I;

**TA II** terminal apophysis II;

**TAA** terminal apophysis appendix;

**TE** tegular extension.

The distribution map was generated with ArcGIS v. 10.5 (Environmental Systems Research Institute, Inc.).

## ﻿Results and discussion

Based on traditional morphological characters and experience (matching of males and females we had hypothesized mainly on the basis of co-occurrence and compatibility of epigynes with male pedipalpal structures), all examined materials could be identified as at least two morphospecies: one belongs to an undescribed species new to science: *L. guiyang* sp. nov.; the other one was identified as *L. fusiformis* (Grasshoff 1970; Chang and Tso 2004; Tanikawa et al. 2023). However, some morphological variation is exhibited in females of *L. guiyang* sp. nov. Five molecular species delimitation methods were employed to test the validity of the morphology-based identification of the three *Lipocrea* species and the accuracy of the proposed sex matching.

**Figure 4.**
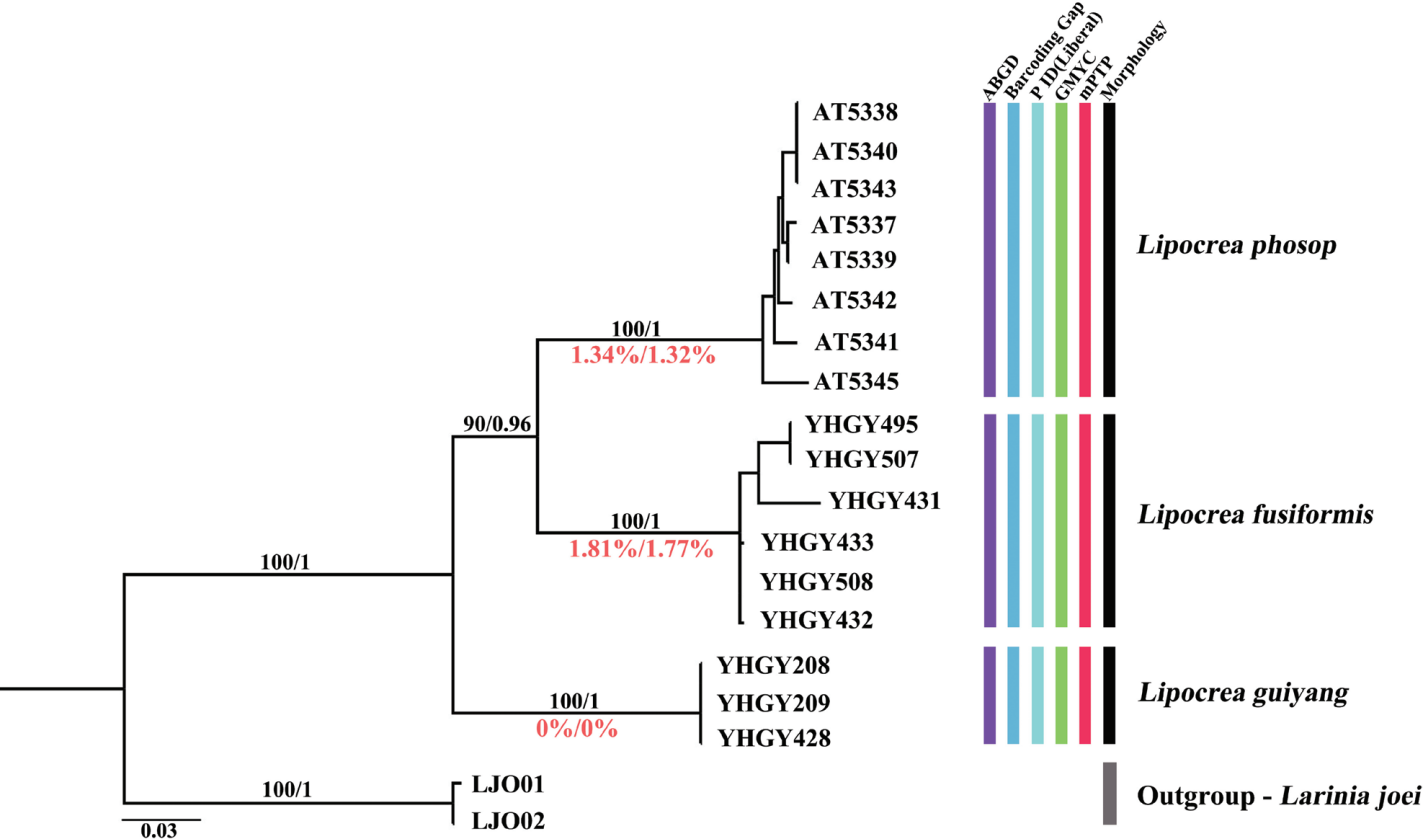
Bayesian CO1 gene tree for 17 terminals of *Lipocrea*, with the results of three different species delimitation approaches. Numbers above branches show bootstrap supports and posterior probability (black), and values above show mean intraspecific (red), calculated as *Lipocrea* two-parameter (K2P)/uncorrected *p*-distance. Species names (for species codes, see Table 1) according to consensus results of species delimitation approaches.

The COI matrix of 17 *Lipocrea* individuals analyzed in this study had a sequence length of 629 bp, with 132 variable and 116 parsimony-informative sites. For COI, phylogenetic inference from BI and ML analyses yielded similar topologies with high support (Fig. 4; posterior probability, PP = 1; bootstrap value, BS = 100). The trees clearly divided the samples into four deeply divergent clades (Fig. 4).

**Figure 5.**
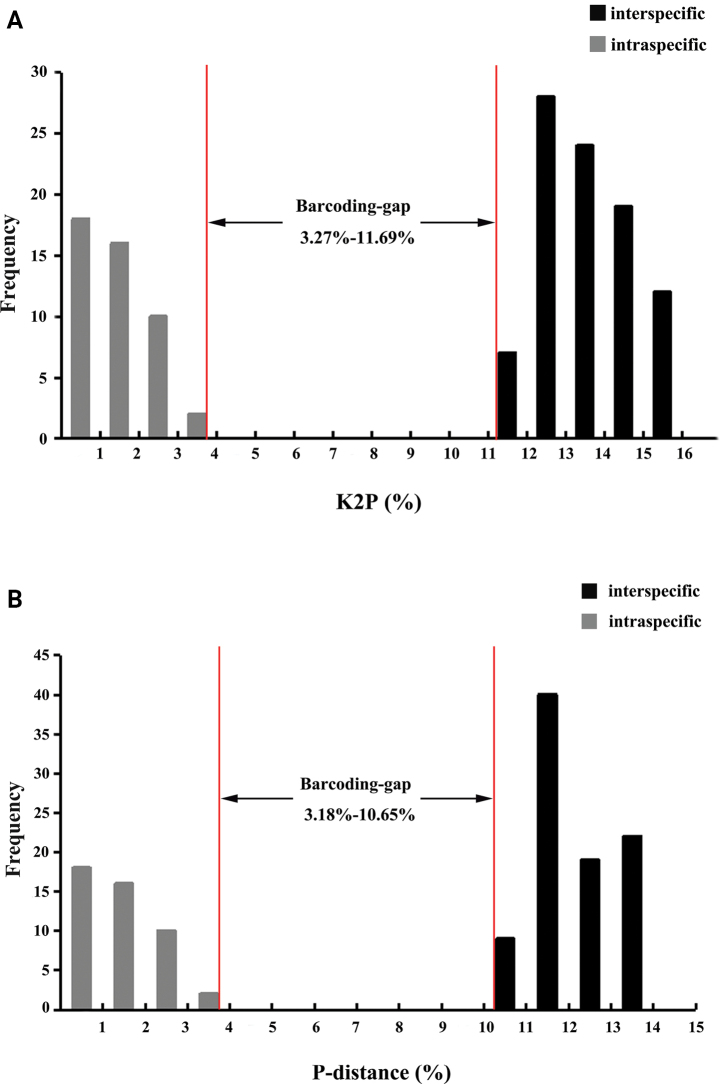
DNA barcoding gap for *Lipocrea*. Histograms show the division of intraspecific (gray) and interspecific (black) CO1 sequence variation based on the Kimura two-parameter (K2P) (A) and uncorrected *p*-distance (B).

When considering three species of *Lipocrea*, interspecific distances were higher than intraspecific distances. Interspecific distances range from 3.27 to 11.69% for K2P (Fig. 5A) and from 3.18 to 10.65% for uncorrected *p*-distance (Fig. 5B). The lowest mean interspecific distance was 12.75% / 11.51% (K2P / uncorrected *p*-distance) found between *L. phosop* and *L. fusiformis*, and the highest mean intraspecific distance (1.81% / 1.77% K2P / uncorrected *p*-distance) was estimated for *L. fusiformis*. The barcoding gap range identified three species, one of which is new (Fig. 4).

The ABGD results varied based on different parameter combinations of both the initial and recursive partitions. Based on the barcoding gap results, a distinct gap was observed among the three *Lipocrea* species, demonstrating substantial genetic divergence. Therefore, the value of X=2 was selected as the relative gap width (Table 2). The initial three species partition was consistent with the three morphospecies, while the recursive partition regime yielded more species (Fig. 4; Table 2). However, all analyses under different assumptions revealed three hypothetical species which is entirely consistent with the morphological identification (Fig. 4).

**Table 2.** Results of the automatic barcode gap discovery (ABGD) analysis.

Substitution model	Pmin/Pmax	X	Partition	Prior intraspecific divergence (P)									
				0.001	0.0017	0.0028	0.0046	0.0077	0.0129	0.0215	0.0359	0.0599	0.1000
JC	0.001/0.1	1.5	Initial	3	3	3	3	3	3	3	3	3	3
			Recursive	9	9	9	9	6	5	3	3	3	3
K2P	0.001/0.1	1.5	Initial	3	3	3	3	3	3	3	3	3	3
			Recursive	9	9	9	9	6	5	3	3	3	3
Simple	0.001/0.1	1.5	Initial	5	5	5	5	3	3	3			
			Recursive	6	6	6	5	4	3	3			
JC	0.001/0.1	2	Initial	3	3	3	3	3	3	3	3	3	3
			Recursive	9	9	9	9	5	5	3	3	3	3
K2P	0.001/0.1	2	Initial	3	3	3	3	3	3	3	3	3	3
			Recursive	9	9	9	9	5	5	3	3	3	3
Simple	0.001/0.1	2	Initial	3	3	3	3	3	3	3			
			Recursive	6	5	5	4	3	3	3			
				0.0001	0.0002	0.0005	0.0013	0.0029	0.0068	0.0159	0.0860		
JC	0.0001/0.2	1.5	Initial	3	3	3	3	3	3	3	3		
			Recursive	9	9	9	9	9	6	5	3		
K2P	0.0001/0.2	1.5	Initial	3	3	3	3	3	3	3	3		
			Recursive	9	9	9	9	9	6	5	3		
Simple	0.0001/0.2	1.5	Initial	5	5	5	5	5	3	3			
			Recursive	6	6	6	6	6	4	3			
JC	0.0001/0.2	2	Initial	3	3	3	3	3	3	3	3		
			Recursive	9	9	9	9	9	5	5	3		
K2P	0.0001/0.2	2	Initial	3	3	3	3	3	3	3	3		
			Recursive	9	9	9	9	9	5	5	3		
Simple	0.0001/0.2	2	Initial	3	3	3	3	3	3	3			
			Recursive	6	6	6	6	5	3	3			

JC, Jukes-Cantor substitution model; K2P, Kimura two-parameter substitution model; Simple, p-distance; X, relative gap width.

Our P ID (Liberal) results revealed high P ID (Liberal) values > 0.95 (0.86–1.0) (Table 3), thereby also supporting the taxonomy of three putative species (Fig. 4).

**Table 3.** Summary of the mean intraspecific and closest interspecific genetic distance, the mean probability with 95% confidence interval, and the intra/interspecific ratio for the three putative species of *Lipocrea*.

Putative species	Intraspecific K2P/p-distance	Closest interspecific K2P/p-distance	P ID (Liberal)	Closest P ID (Liberal) species	Intra/Inter
*L. guiyang* sp. nov.	0/0	0.1349/0.1219	1.00 (0.86, 1.0)	*L. fusiformis*	0.02
*L. phosop*	0.0134/0.0132	0.1275/0.1151	0.97 (0.90, 1.0)	*L. fusiformis*	0.12
*L. fusiformis*	0.0181/0.0177	0.1275/0.1151	0.96 (0.86, 1.0)	*L. phosop*	0.14

The results of our mPTP analysis indicated that, when only monophyletic species are considered, our three hypothetical species were identified as anticipated (Fig. 4). The mPTP model strongly supports three species: *L. guiyang* sp. nov., *L. phosop* and *L. fusiformis* (PP = 1.0).

GMYC produced the same results as the four methods mentioned above (Fig. 4). The single-threshold model GMYC resulted in three clusters (confidence intervals: 1–5) and three entities (confidence intervals: 1–17) (Table 4).

**Table 4.** Results of the general mixed Yule-coalescent (GMYC) analyses (***P < 0.001).

Analysis	Clusters (CI)	Entities (EI)	Likelihood (null)	Likelihood (GMYC)	Likelihood ratio	Threshold
Single	3(1–5)	3(1–17)	96.89582	98.26415	2.736648	-0.017105236

In conclusion, all analyses support three morphological species, with terminals within the same clade being conspecific. In *L. guiyang* sp. nov., the results from five molecular species delimitation analyses indicate that, despite considerable morphological variation among individuals, they should be classified as the same species: (1) Both the ABGD and GMYC methods, whether using multiple parameter setting s or visualizing the data tree, support a single species (Fig. 4, Tables 2, 4); (2) these results are also supported by both the P ID (Liberal) and mPTP methods, with a P ID (Liberal) value of 1.00 (0.86, 1.0) (> 95%) (Table 3) and a posterior probability of one in mPTP; (3) our barcoding gap results (3.27% – 11.69% / 3.18% – 10.65% / K2P/uncorrected p-distance) further support that the average genetic distance (0% / 0% K2P/uncorrected p-distance) of *L. guiyang* sp. nov. represents the intraspecific genetic distance (Figs 4, 5). After careful re-examination of all specimens, the variation is concluded to be intraspecific differences. We also confirm sex pairing accuracy of both morphospecies based on an integrated morphological and molecular approach, including *L. guiyang* sp. nov. The molecular and morphological identifications are therefore congruent.

As can be concluded from the above, when identifying certain species of the genus *Lipocrea*, relying solely on morphological characters may lead to over-splitting of species. Nevertheless, our results indicate that even single-locus analyses based on the COI barcodes, when integrated with morphological data and collection experience, may provide sufficiently reliable species delimitation.

### ﻿Taxonomic accounts

**Family Araneidae Clerck, 1757**

#### ﻿Genus Lipocrea

Taxon classificationAnimaliaAraneaeAraneidae

Thorell, 1878

540FDC1F-ABAD-5393-91BC-A719732BC7B9

##### Type species.

*Meta fusiformis* Thorell, 1877, from Indonesia (Sulawesi), India, Myanmar, Vietnam, Japan, Bangladesh, Philippines, Thailand, China, New Guinea, Australia (Grasshoff 1970; Tanikawa 1989; Okuma et al. 1993; Barrion and Litsinger 1995; Chang and Tso 2004).

##### Amended diagnosis.

*Lipocrea* differs from *Larinia* sensu stricto by: the broad epigynal base with auricular lateral edges and a bipartite posterior margin (vs epigynal base not widening and posterior margin not bipartite) (cf. Figs 10C, 14D and Grasshoff 1970: fig. 6d; Gaymard and Lecigne 2018: fig. 9B; Morano 2023: figs 81, 82); conductor broadly fused with the tegular extension, forming a continuous structure (conductor separated from tegular extension) (cf. Figs 8A–C, 9A–C, E, 13B, C and Grasshoff 1970: fig. 6b, c, Gaymard and Lecigne 2018: fig. 9D, E, Morano 2023: fig. 80); median apophysis with a conspicuous spine-like or hook-shaped process with a sharp apex (vs. process absent) (cf. Figs 8A–C, 9A–C, E, 13A–C and Grasshoff 1970: fig. 6b, c, Alioua et al. 2020: 2, fig. 9, Gaymard and Lecigne 2018: fig. 9D, E, Morano 2023: fig. 80); radix with a keel-like ridge (vs no ridge) (cf. Figs 8A, C, 9A, C, E, 13A, C and Grasshoff 1970: fig. 6a, b, Alioua et al. 2020: 2, fig. 9).

**Figure 6.**
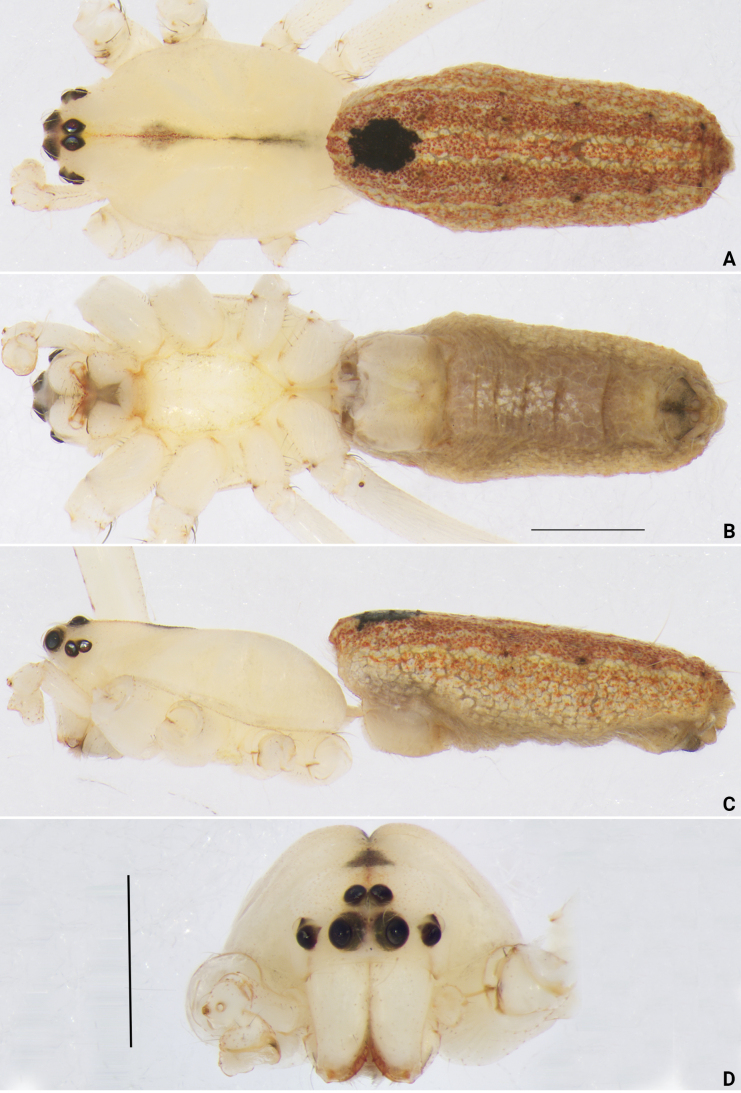
*Lipocrea guiyang* J. Zhang, Yu & Mi, sp. nov., male holotype (YHGY208), habitus (A–C) and frontal view of cephalothorax (D). A. Dorsal; B. Ventral; C. Lateral; D. Male. Scale bars: 1 mm (A–C).

##### Composition and distribution.

The genus *Lipocrea* currently comprises five species (WSC 2025): *L. diluta* Thorell, 1887, distributed from Myanmar to Indonesia; *L. epeiroides* (O. Pickard-Cambridge, 1872), occurring from Spain and Italy (including Sardinia and Sicily) to Malta, Cyprus, Turkey, Israel, Yemen, and India; *L. fusiformis* (Thorell, 1877), with a distribution extending from India to Japan, the Philippines, and Indonesia (Sulawesi); *L. longissima* (Simon, 1881), found throughout central, eastern, and southern Africa; and *L. phosop* (Tanikawa, Into & Petcharad, 2023), currently known only from Thailand. This paper reports the sixth member of the genus, *L. guiyang* sp. nov., currently endemic to Guiyang City, China.

Based on preliminary molecular phylogenetic results (Suppl. material 1: fig. S1) and morphological evidence (see diagnosis above), *L. fusiformis*, *L. guiyang* sp. nov., and *L. phosop* are confidently placed in the genus *Lipocrea* (Figs 8–10B–F, 11, 13, 14; Tanikawa et al. 2023 figs 3–14). In addition, although not included in our molecular analyses, *L. epeiroides* (O. Pickard-Cambridge, 1872) and *L. longissima* (Simon, 1881) exhibit characteristics typical of *Lipocrea* and are thus justifiably assigned to this genus (Bosmans and Colombo 2015: figs 43–48; Grasshoff 1970: figs 12a, b, 14a–c). However, due to the absence of a spine-like or hook-shaped process on the median apophysis, a keel-like ridge on the radix, and a broad epigynal base in *L. diluta* (Grasshoff 1970: fig. 17a–c), there remains considerable uncertainty regarding the placement of this species within *Lipocrea*.

#### ﻿ Lipocrea guiyang

Taxon classificationAnimaliaAraneaeAraneidae

J. Zhang, Yu & Mi sp. nov.

958E9D71-6833-5B8E-8ABF-5E6B0CE739CE

https://zoobank.org/200B8EC9-2032-49B4-9483-91D65AC0887D

Figs 1, 2, 6, 7, 8, 9, 10, 11

##### Type material.

***Holotype*** • ♂ (YHGY208), China: Guizhou Prov.: Guiyang City, Kaiyang Co., Nanjiang Grand Canyon, 26.94°N, 106.97°E, c. 861 m, by hand, 7 VI 2022, H. Yu & Q. Lu leg. ***Paratypes***: • 2♀ (YHGY209, YHGY428), same data as holotype.

##### Other material examined.

• 1♂1♀ (YHGY218, YHGY318), same data as holotype.

##### Diagnosis.

Males of *L. guiyang* sp. nov. can be easily distinguished from all other congeners with the exception of *L. phosop* by having a bifurcate terminal apophysis (*TA*); a terminal apophysis appendix (*TAA*) that is relatively short, with its apex not extending beyond the apex of the *TA* and directed distally; and a relatively long conductor (*C*) that is nearly as long as the tegular extension (*TE*) (Figs 8A–C, 9A–E; Tanikawa et al. 2023: figs 3–5, 9–11) (*TA* not bifurcate, *TAA* tongue-shaped, wrapping around *TA*, apex directed proximally, *C* rostrate and distinctly shorter than *TE*, such as in *L. epeiroides*, *L. fusiformis* and *L. longissima* as in Fig. 13A–C, Levy 1986: figs 32–34, Tanikawa 2009: figs 191, 192, Grasshoff 1970: figs 12a, b,15a, b) but can be easily differentiated from *L. phosop* by: (1) *TA* distinctly bifurcating into two apophyses (vs slightly bifurcate and not forming two processes) (cf. Figs 8B, C, 9A, B, D, E and Tanikawa et al. 2023: figs 3–5, 9–11); (2) *TAA* digitiform (vs papilliform) (cf. Figs 8B, C, 9B, C, E and Tanikawa et al. 2023: figs 3, 9–11); (3) process of median apophysis (*HP*) hook-shaped, distinctly curved (vs nearly triangular) (cf. Figs 8A–C, 9A–C, E and Tanikawa et al. 2023: figs 3–5, 9–11); (4) *C* shaped like a ox horn (vs finger-shaped) (cf. Figs 8A–C, 9A–C, E and Tanikawa et al. 2023: figs 3–5, 9–11). Females of *L. guiyang* sp. nov. also resemble those of *L. phosop* by the presence of a knob-shaped projection (*KP*) (epigyne with a scape instead of a *KP* in all other congeners, such as *L. fusiformis*; as in Fig. 14A–D), but it can be easily differentiated from *L. phosop* by the following features: (1) epigyne nearly trapeziform or disc-shaped in ventral view (vs inverted heart shape) (cf. Figs 10C, 11A, C and Tanikawa et al. 2023: figs 6, 12); (2) *KP* partly membranous and translucent (vs *KP* more sclerotized, non-transparent) (cf. Fig. 10C and Tanikawa et al. 2023: figs 6, 12); (3) copulatory openings (*CO*) located on comma-shaped or circular windows, with the posterior margin clearly separated from the posterior margin of epigyne (vs windows shaped like a horizontally oriented check mark, posterior margin of *CO* close to posterior margin of epigyne) (cf. Figs 10C, 11A, C and Tanikawa et al. 2023: figs 6, 12).

##### Description.

**Male (holotype, YHGY208). *Measurements*.** Total length 6.09. Carapace 2.70 long, 1.77 wide. Abdomen 3.55 long, 1.52 wide. Sternum 1.30 long and 0.76 wide. Labium 0.19 long and 0.39 wide. Endites 0.55 long and 0.35 wide. Clypeus height 0.10. Both margins of chelicerae with four teeth. Eye sizes and interdistances: AME 0.17, ALE 0.12, PME 0.14, PLE 0.11, AME–AME 0.20, ALE–AME 0.12, PME–PME 0.03, PME–PLE 0.26. MOQ 0.43 long, anterior width 0.47, posterior width 0.28. Leg measurements: I missing, II 12.63 (3.07, 4.55, 3.91, 1.10), III 6.22 (2.09, 2.03, 1.45, 0.65), IV 10.53 (3.11, 3.53, 3.08, 0.81).

***Habitus* (Fig. 6A–D).** Carapace nearly oval, basically yellowish white, with a narrow red midline extending from just behind the PME almost to the black fovea; a faint dark spot present approximately at the midpoint of the midline; pars cephalica distinctly narrowed; cervical groove and radial grooves invisible; tegument smooth. AER distinctly recurved, PER nearly as wide as AER, almost straight in dorsal view. Sternum bright yellow, shield shaped; anterior margin nearly straight, posterior region strongly protruding between coxae III. Chelicerae colored as carapace, with reddish fangs. Labium and endites colored as carapace; labium nearly triangular, concave laterally; endites depressed posteriorly, slightly convergent anteriorly, with dense scopulae on inner margin. Legs uniformly colored as carapace. Abdomen elongate-oval; dorsum reddish brown, with a sword-shaped median band extending along its entire length, bordered with yellow lines and bearing a prominent large black spot anteriorly; laterally with two distinct yellow longitudinal lines, each line accompanied by approximately four small black spots; venter grayish, without distinct pattern; spinnerets yellow.

***Palp* (Figs 8A–D, 9A–E).** Cymbium (*Cy*) navicular, ~2.2× longer than wide, dorsally with sparse, long setae (all detached in ethanol), basoretrolaterally with a thumb-like paracymbium (*Pc*). *Pc* moderately large, about 1/5 length of cymbium, apex blunt, slightly curved and pointing retrolatero-distally. Tegulum (*T*) disc-shaped, slightly wider than cymbium, with distinct sperm duct along anterior margin, proximally covered by broad subtegulum (*St*). Tegular extension (*TE*) laminar, extending dorsally, almost completely concealed by conductor (*C*) in ventral view. *St* ~1/2 cymbium length, partly membranous, surface wrinkled and ribbed, with numerous diagonal ridges. Radix (*R*) leaf shaped, ~½ the width of the subtegulum length, distally with a triangular apophysis (*RA*). *RA* hyaline, nearly as long as radix, apex sharp and pointing distally. Median apophysis (*MA*) heavily sclerotized, located prolaterally to tegulum, consisting of a broad base and a hook-shaped process (*HP*); base navicular, ~2/5 the width of the subtegulum in length; *HP* nearly as long as base, apex sharp, distinctly curved and pointing retrolaterally. Terminal apophysis (*TA*) hidden behind tegulum, extending distally, apex surpassing the tegulum and bifurcating into two apophyses, forming a C-shape in anterior view; both terminal apophysis I (*TA I*) and terminal apophysis II (*TA II*) heavily sclerotized, with blunt apices pointing prolaterally; *TA I* relatively large, its length nearly equal to the width of the tegulum; *TA II* smaller and humble, ~½ the length of *TAI*. Terminal apophysis appendix (*TAA*) membranous, digitiform, accompanied by terminal apophysis, hidden behind tegulum, extending distally. *C* originating from dorsal-anterior portion of tegulum, proximally fused to weakly sclerotized *TE*; tip distinctly curved, shaped like an ox horn, with a sharp apex pointing dorso-distally. Embolus (*Em*) spine-shaped, nearly as long as the hook-shaped process of the median apophysis, originating centrally in anterior view, extending distally, surrounded by the *RA*, *MA*, *TA*, and *C*.

**Female (YHGY428).** Total length 7.30. Carapace 2.83 long, 1.81 wide. Abdomen 4.96 long, 2.67 wide. Sternum 1.25 long and 0.84 wide. Labium 0.28 long and 0.49 wide. Endites 0.61 long and 0.43wide. Clypeus height 0.10. Both margins of chelicerae with four teeth. Eye sizes and interdistances: AME 0.15, ALE 0.13, PME 0.13, PLE 0.11, AME–AME 0.23, ALE–AME 0.19, PME–PME 0.02, PME–PLE 0.35. MOQ 0.44 long, anterior width 0.48, posterior width 0.27. Leg measurements: I 14.53 (3.33, 5.36, 4.54, 1.30), II 13.15 (3.26, 4.89, 3.86, 1.14), III 6.96 (2.31, 2.27, 1.58, 0.80), IV 11.80 (3.28, 4.39, 3.14, 0.99).

**Figure 7.**
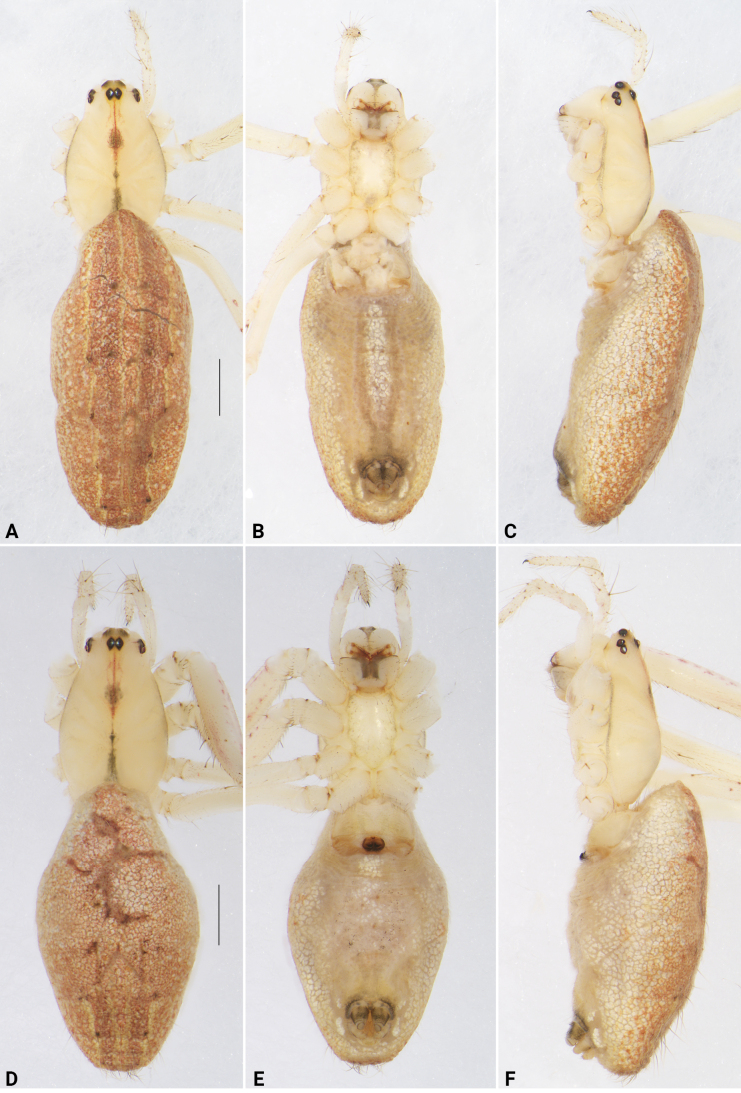
*Lipocrea guiyang* J. Zhang, Yu & Mi, sp. nov., female paratypes, YHGY428 (A–C) and YHGY209 (D–F), habitus. A, D. Dorsal; B, E. Ventral; C, F. Lateral. Scale bars: 1 mm.

***Habitus* (Fig. 7A–C, 10A).** Similar to males, but the dorsum of the abdomen lacks the prominent anterior black spot.

**Figure 8.**
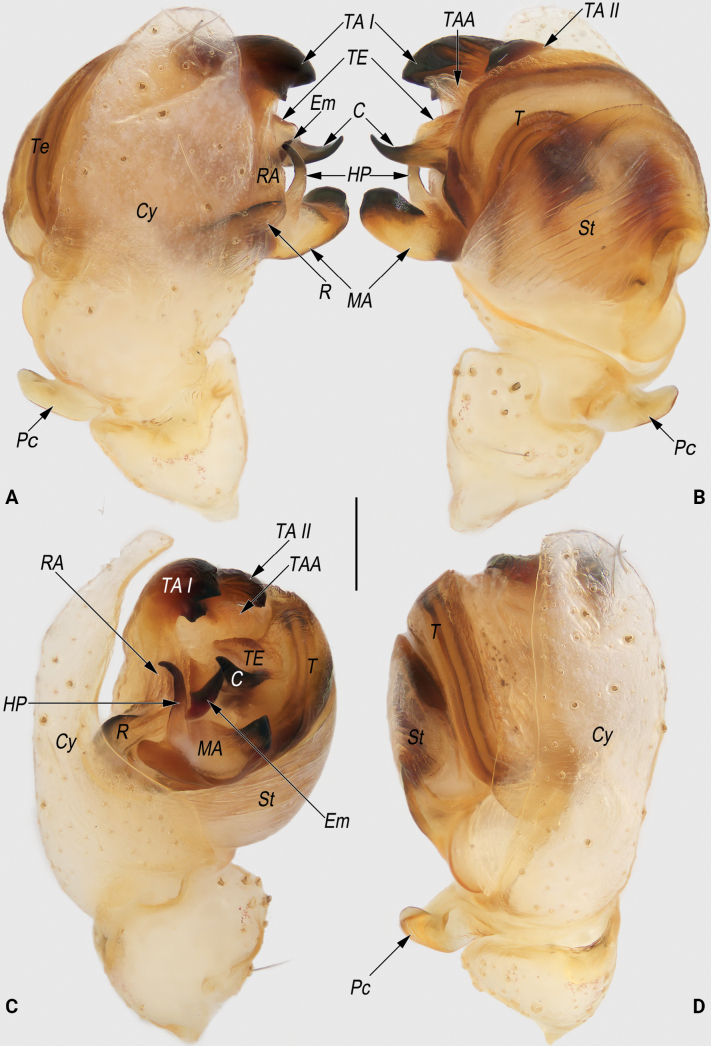
Male palp of the holotype of *Lipocrea guiyang* J. Zhang, Yu & Mi, sp. nov. A. Dorsal; B. Ventral; C. Prolateral; D. Retrolateral. Abbreviations: C = conductor; Cy = cymbium; Em = embolus; HP = hook-shaped process of MA; MA = median apophysis; Pc = paracymbium; R = radix; RA = radix apophysis; St = subtegulum; T = tegulum; TA I = terminal apophysis I; TA II = terminal apophysis II; TAA = terminal apophysis appendix; TE = tegular extension. Scale bar: 0.2 mm.

**Figure 9.**
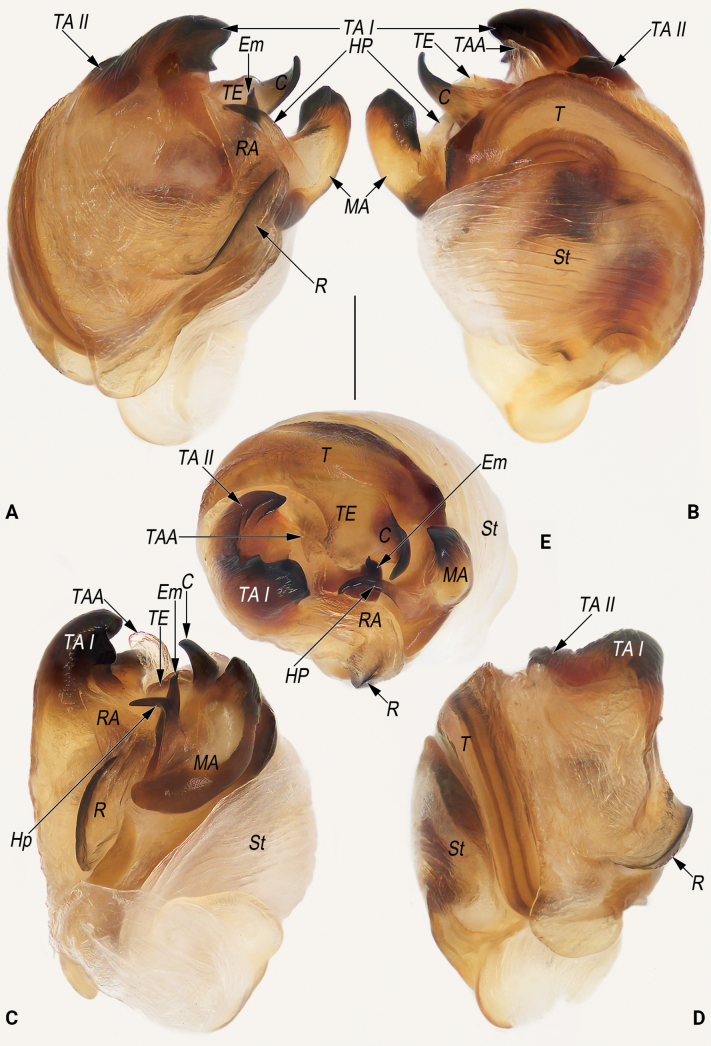
Male palpal bulb of the holotype of *Lipocrea guiyang* J. Zhang, Yu & Mi, sp. nov. A. Dorsal; B. Ventral; C. Prolateral; D. Retrolateral; E. Anterior. Abbreviations: C = conductor; Em = embolus; HP = hook-shaped process of MA; MA = median apophysis; R = radix; RA = radix apophysis; St = subtegulum; T = tegulum; TA I = terminal apophysis I; TA II = terminal apophysis II; TAA = terminal apophysis appendix; TE = tegular extension. Scale bar: 0.2 mm.

**Figure 10.**
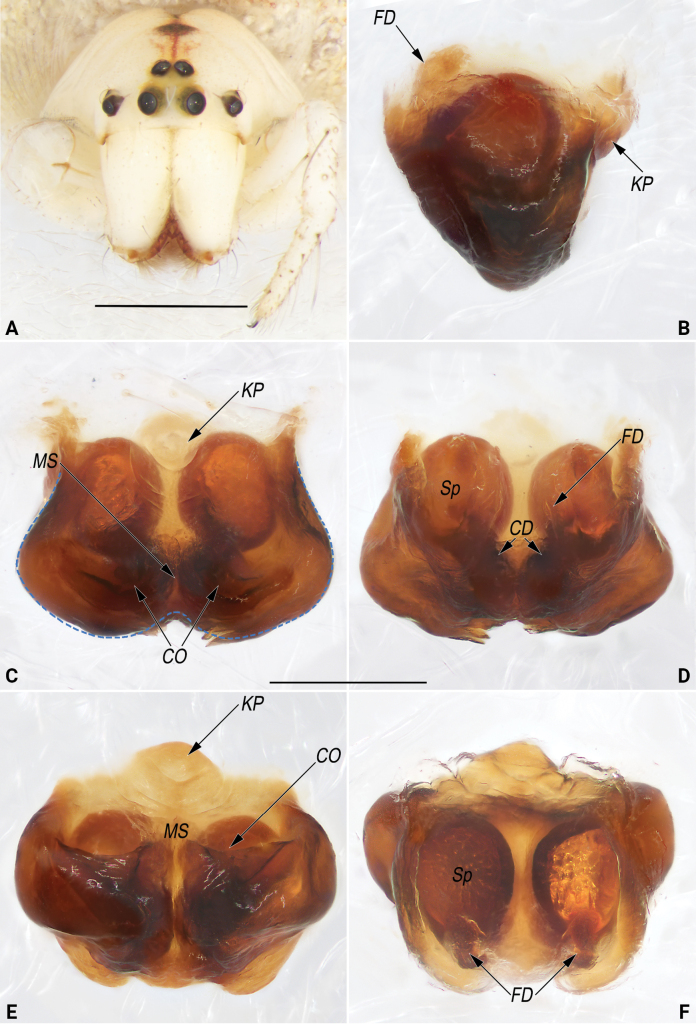
Frontal view of cephalothorax (A.) and macerated epigyne (B–F) of the paratype (YHGY428) of *Lipocrea guiyang* J. Zhang, Yu & Mi, sp. nov. A. Female; B. Lateral; C. Ventral (blue dashed line showing the lateral and posterior margins of the epigynal base); D. Dorsal; E. Ventro-posterior; F. Dorso-anterior. Abbreviations: CD = copulatory duct; CO = copulatory opening; FD = fertilization duct; KP = knob-like projection; MS = median septum; Sp = spermatheca. Scale bars: 1 mm (A), 0.2 mm (B–F).

**Figure 11.**
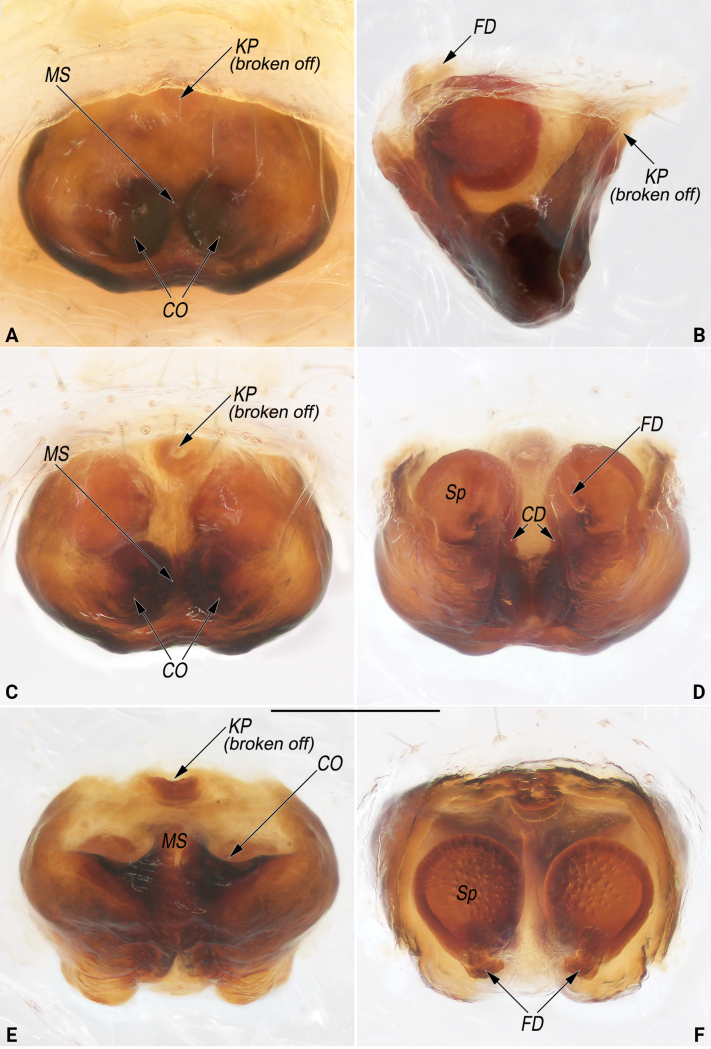
Epigyne of the paratype (YHGY209) of *Lipocrea guiyang* J. Zhang, Yu & Mi, sp. nov. A. Intact, ventral; B. Macerated, lateral; C. Macerated, ventral; D. Macerated, dorsal; E. Macerated, ventro-posterior; F. Macerated, dorso-anterior. Abbreviations: CD = copulatory duct; CO = copulatory opening; FD = fertilization duct; KP = knob-like projection; MS = median septum; Sp = spermatheca. Scale bar: 0.2 mm.

***Genitalia* (Fig. 10B–F).** Epigyne strongly sclerotized with large postero-lateral lobes, distinctly wider than long, nearly trapeziform in ventral view and inverted triangular in lateral view. Knob-shaped projection (*KP*) represented by a small, partly membranous tubercle, located at anterior portion of epigynal plate. Copulatory openings (*CO*) large, located on the comma-shaped window (or pockets with chitinous posterior margins) is at the postero-lateral portion of the epigynal plate, separated by indistinct median septum (*MS*). Copulatory ducts (*CD*) short, diverging and ascending obliquely, forming V-shaped course in dorsal view, finally entering anteriorly located spermathecae. Spermathecae (*Sp*) oval, ~1.2× longer than wide, relatively large, ~2/3 of epigyne length; two spermathecae close together, separated by ~2/3 of their width. Fertilization ducts (*FD*) membranous, relatively long, ~2/3 of spermathecae length, located on dorsal-basal surface of spermathecae.

##### Distribution.

Known only from the type locality (Fig. 1).

##### Etymology.

The specific epithet is derived from the name of the type locality; noun in apposition.

##### Comments.

In spite of the stable morphology of the male palp and the consistent coloration of the male habitus, considerable morphological variation is observed among female individuals, primarily related to epigynal structures. These variations involve features such as the presence or absence of a knob-shaped projection (*KP*), the shape of the copulatory openings (*CO*), and whether the median septum (*MS*) is distinct or indistinct. For example, in some females (e.g., YHGY428, as in Fig. 10B, C, E), the *KP* is distinct, the *CO* is situated on a comma-shaped window (or within pockets with chitinous posterior margins), and the *MS* is indistinct. In contrast, in other individuals (e.g., YHGY209, as in Fig. 11A–C, E), the *KP* is broken off, the *CO* is positioned on a nearly circular window (or within pockets bordered anteriorly, internally, and posteriorly), and the *MS* is distinct. In addition, some variation related to the abdominal pattern is also observed: the dorsum of the abdomen bears a median band extending along its entire length in some individuals (e.g., YHGY428), whereas in others (e.g., YHGY209), the median band is restricted to the posterior quarter of the dorsum (cf. Fig. 7A and Fig. 7D). However, the morphological variation was determined to be intraspecific variation based on the molecular species delimitation analysis.

#### ﻿ Lipocrea fusiformis

Taxon classificationAnimaliaAraneaeAraneidae

(Thorell, 1877)

79C3FD6A-E5F2-5664-B148-B1F22769C556

**Figure 12.**
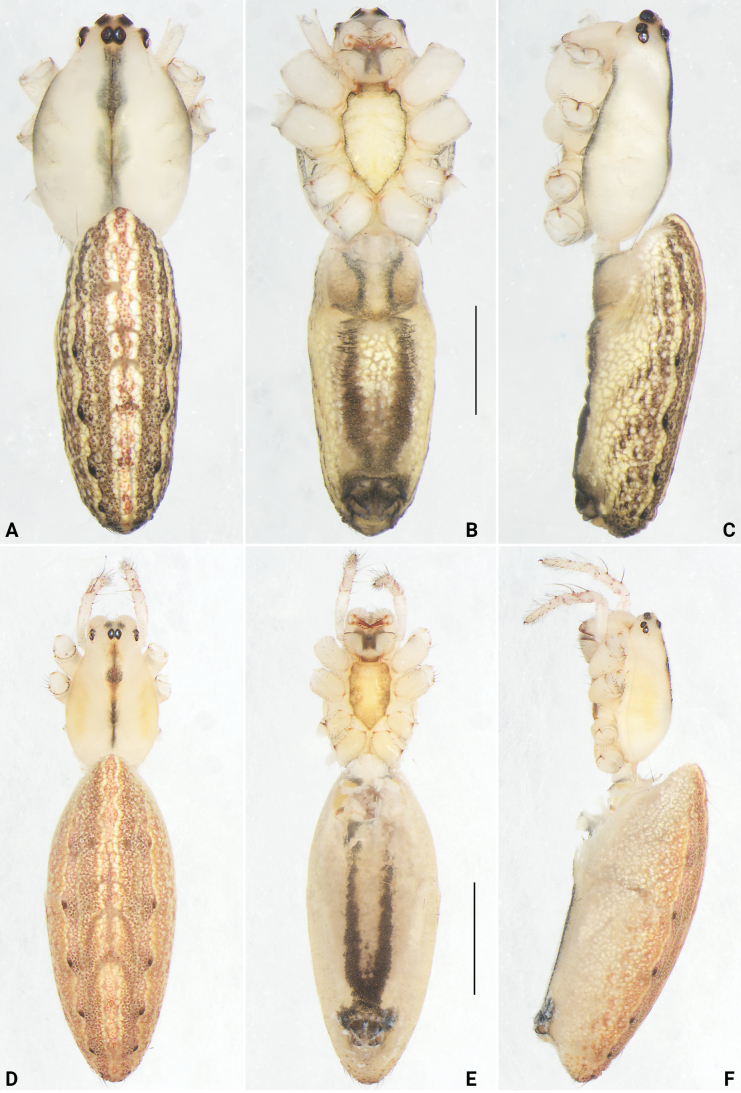
*Lipocrea fusiformis* (Thorell, 1877), male (YHGY431, A–C) and female (YHGY432, D–F), habitus. A, D. Dorsal; B, E. Ventral; C, F. Lateral. Scale bars: 1 mm (A–C), 2 mm (D–F).

**Figure 13.**
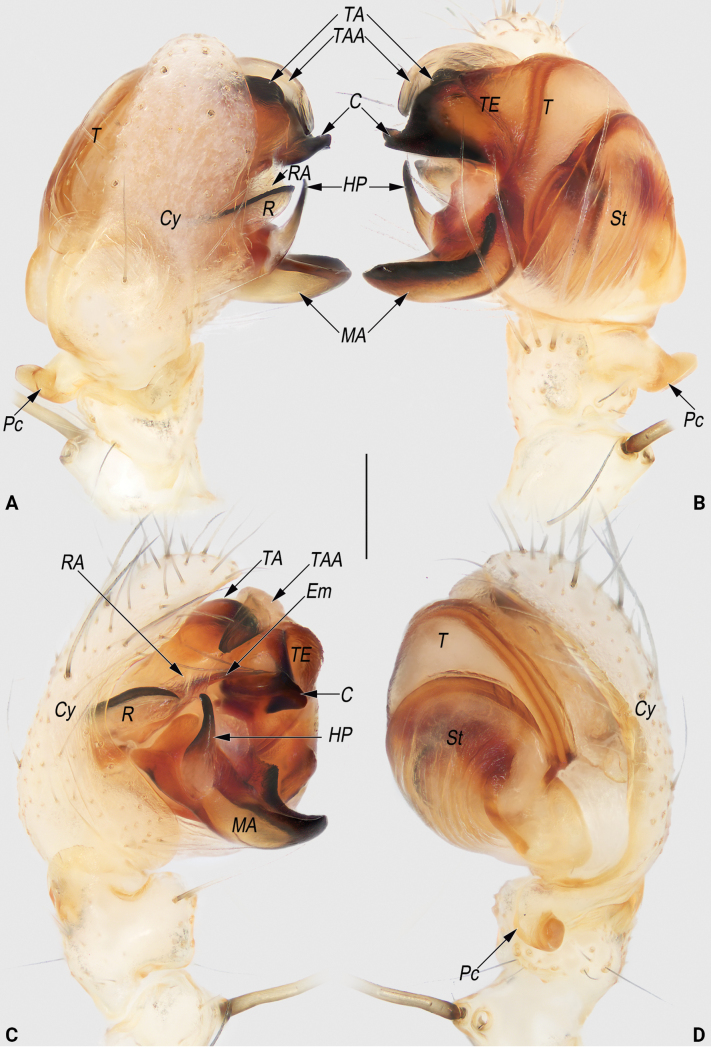
Male palp of *Lipocrea fusiformis* (Thorell, 1877). A. Dorsal; B. Ventral; C. Prolateral; D. Retrolateral. Abbreviations: C = conductor; Cy = cymbium; Em = embolus; HP = hook-shaped process of MA; MA = median apophysis; Pc = paracymbium; R = radix; RA = radix apophysis; St = subtegulum; T = tegulum; TA = terminal apophysis; TAA = terminal apophysis appendix; TE = tegular extension. Scale bar: 0.2 mm.

**Figure 14:**
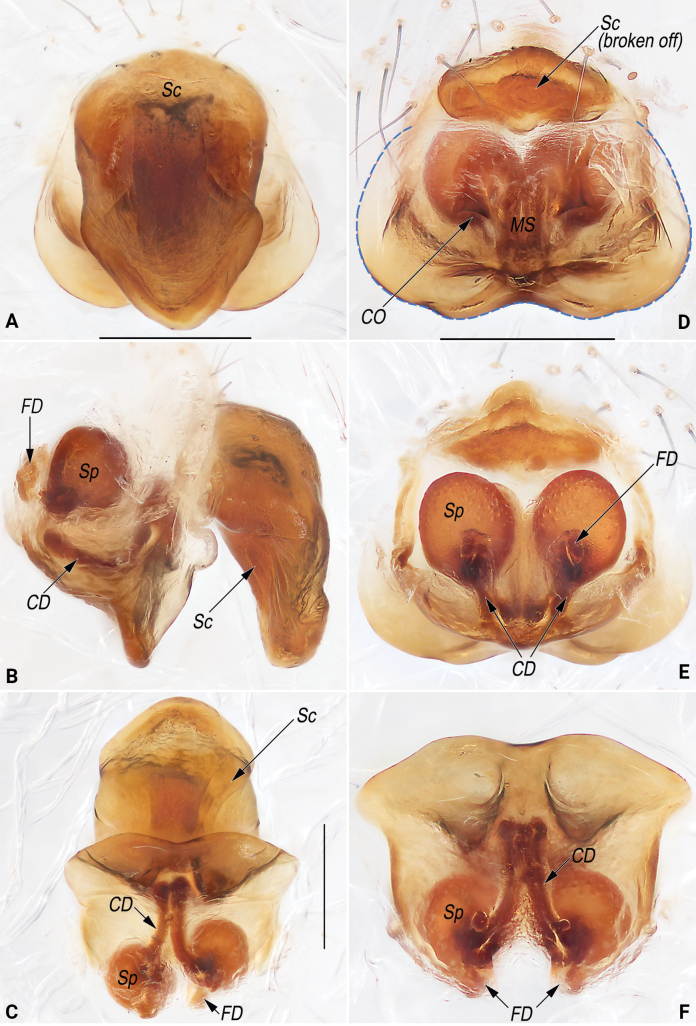
Macerated epigynes of females of *Lipocrea fusiformis* (Thorell, 1877), YHGY432 (A–C) and YHGY507 (D–F). A. Ventral, with scape; B. Lateral, with scape; C. Ventro-posterior, with scape; D. Ventral, without scape (blue dashed line showing the lateral and posterior margins of the epigynal base); E. Dorsal, without scape; F. Ventro-posterior, without scape. Abbreviations: CD = copulatory duct; CO = copulatory opening; FD = fertilization duct; MS = median septum; Sc = scape; Sp = spermatheca. Scale bars: 0.2 mm (A, B, D–F).

Figs 1, 3, 12, 13, 14

Meta fusiformis Thorell, 1877: 431 (♀).
Lipocrea fusiformis : Thorell 1878: 6.
Larinia quadrinotata Simon, 1889: 340 (juv.); Simon 1909: 105 (♀).
Larinia lutescens Thorell, 1898: 342 (♂♀).
Larinopa fusiformis : Grasshoff 1970: 231, figs 15a, b, 16a–e (♂♀, transfer from Larinia, synonym of Larinia lutescens and L. quadrinotata); Tanikawa 1989: 35, figs 8–14 (♂♀); Chang and Tso 2004: 28, figs 5–8 (♂♀); Tanikawa et al. 2023: 56, figs 15–18.

##### Note.

For full list of taxonomic references, see WSC (2025).

##### Material examined.

• 1♂, 3♀ (YHGY432, YHGY495, YHGY507, YHGY508), China: Guizhou Prov.: Guiyang City, Huaxi District, University Town, 26.38°N, 106.65°E, c. 1173 m, by beating, 7 VII 2022, Q. Jiang & Q. Du leg; • 1♂, 1♀ (YHGY431, YHGY433), Guiyang City, Huaxi District, Dangwu Town, 26.38°N, 106.60°E, c. 1138 m, by hand, 19 V 2022, H. Yu & Q. Lu leg; Guiyang City, Kaiyang Co., Nanjiang Grand Canyon, 26.94°N, 106.97°E, c. 861 m, by hand, 7 VI 2022, H. Yu & Q. Lu leg.

##### Diagnosis and description.

See Tanikawa (1989). Living specimens as in Fig. 3A–C, male habitus as in Fig. 12A–C, male palp as in Fig. 13A–D, female habitus as in Fig. 12D–F, genitalia as in Fig. 14A–F.

##### Distribution.

China (Guizhou, new record for mainland China; Taiwan), India, Burma, Vietnam, Thailand, Japan, Philippines, Sulawesi, New Guinea, Australia.

##### Comments.

Among all examined female specimens, the epigynal scape is broken off in some individuals (e.g., YHGY507, as in Fig. 14D–F), but it remains intact in others (e.g., YHGY432, as in Fig. 14A–C). Aside from this difference, all other morphological features are consistent. In addition, results from molecular evidence confirm that these specimens belong to the same species (Fig. 4; for details, see the results and discussion section).

## Supplementary Material

XML Treatment for Lipocrea

XML Treatment for Lipocrea guiyang

XML Treatment for Lipocrea fusiformis

## ﻿Additional information

### Use of AI

No use of AI was reported.

### Author ORCIDs

Jianshuang Zhang https://orcid.org/0000-0003-4010-3082

Chengwen Zhang https://orcid.org/0009-0008-1258-5727

Yuanqian Xing https://orcid.org/0000-0002-6278-9778

Hao Yu https://orcid.org/0000-0002-9113-2425

Xiaoqi Mi https://orcid.org/0000-0003-1744-3855

### Data availability

All of the data that support the findings of this study are available in the main text or Supplementary Information.

## Supplementary materials

Supplementary material 1 Test of the monophyly of the genus *Lipocrea* based on available CO1 sequences Data type docx Explanation note **table S1.** Samples used in phylogenetic analyses: species and GenBank accession numbers. **fig. S1.** Maximum-likelihood tree constructed based on the data in table S1. https://binary.pensoft.net/file/1435290This dataset is made available under the Open Database License (http://opendatacommons.org/licenses/odbl/1.0/). The Open Database License (ODbL) is a license agreement intended to allow users to freely share, modify, and use this Dataset while maintaining this same freedom for others, provided that the original source and author(s) are credited.Jianshuang Zhang, Hao Yu, Xiaoqi Mi
