# Supplementary material for: ﻿A survey of the spider genus Lipocrea Thorell, 1878 (Araneae, Araneidae) from Guiyang City, Southwest China: An integrated morphological and molecular approach
Source: Zookeys. 2025 Oct 10;1255:207–37. doi: 10.3897/zookeys.1255.158340 (PMC12534792; doi:10.3897/zookeys.1255.158340)
Supplement: Supplementary material 1 — Test of the monophyly of the genus Lipocrea based on available CO1 sequences [file zookeys-1255-207_article-158340__-s001.docx]

Table S1. Samples used in phylogenetic analyses: species and GenBank accession numbers.

| species | COI | species | COI |
| --- | --- | --- | --- |
| Araneus angulatus UB-MD653 | MW996976 | Araniella displicata 2062 | JN307933 |
| Araneus angulatus UB-MD652 | MW996975 | Araniella displicata 2061 | JN307932 |
| Araneus angulatus UB-MD651 | MW996974 | Araniella cucurbitina MD2406 | MW997013 |
| Araneus ventricosus AVE1 | KY467248 | Araniella cucurbitina MD2407 | MW997014 |
| Araneus ventricosus AVE2 | KY467249 | Araniella cucurbitina MD2408 | MW997015 |
| Araneus ventricosus AVE3 | KY467250 | Araniella alpica UB-MD660 | MW996990 |
| Argiope lobata UOS-00082 | MK154282 | Araniella alpica UB-MD661 | MW996989 |
| Argiope lobata SPD-01276 | MK154255 | Larinia bonneti | MK420119 |
| Argiope aurantia 00001285A | KX817158 | Larinia borealis | KP648467 |
| Argiope aurantia 00001351A | KX817147 | Larinia directa E02 | KM834197 |
| Hypsosinga sanguinea ZMUO368 | MZ608637 | Larinia directa F03 | KM833458 |
| Hypsosinga sanguinea ZMUO369 | MZ608280 | Larinia directa F04 | KM832082 |
| Hypsosinga heri TIS18365 | KY269911 | Larinia directa F02 | JN308599 |
| Hypsosinga heri TIS22272 | KY269702 | Larinia jeskovi | MK420120 |
| Hypsosinga wanica SPD00455 | JN306327 | Larinia joei LJO01 | LC597525 |
| Hypsosinga wanica SPD00362 | JN306254 | Larinia joei LJO02 | LC597526 |
| Lariniaria argiopiformis | JN817161 | Larinia phthisica LPH02 | LC597528 |
| Larinioides cornutus ZMUO88 | MZ608124 | Larinia phthisica LPH01 | LC597527 |
| Larinioides cornutus ZMUO98 | MZ632953 | Larinia phthisica COTSP2 | PV600250 |
| Larinioides sclopetarius C04 | HQ977057 | Larinia phthisica COTSP9 | PV596076 |
| Larinioides sclopetariusGAB093 | GU682444 | Larinia phthisica SPD00268 | JN306172 |
| Lipocrea fusiformis | LC597529 | Larinia phthisica SPD00212 | JN306125 |
| Lipocrea phosop AT5345 | LC756460 | Larinia phthisica SPD00125 | JN306048 |
| Lipocrea phosop AT5342 | LC756459 | Larinia phthisica SPD00084 | HQ991608 |
| Lipocrea phosop AT5341 | LC756458 | Larinia phthisica SPD00080 | HQ991604 |
| Lipocrea phosop AT5340 | LC756457 | Larinia sp 1207 | OR895076 |
| Lipocrea phosop AT5339 | LC756456 | Larinia sp 1205 | OR895074 |
| Lipocrea phosop AT5338 | LC756455 | Larinia sp. 1GAB_PAK | MK154822 |
| Lipocrea phosop AT5337 | LC756454 | Nuctenea umbratica MD1942 | MW998221 |
| Lipocrea phosop AT5343 | LC756453 | Nuctenea umbratica MD1944 | MW998220 |
| YHGY431 Lipocrea fusiformis | PX230061 | Nuctenea silvicultrix ZMUO378 | MZ610833 |
| YHGY432 Lipocrea fusiformis | PX230062 | Nuctenea silvicultrix ZMUO379 | MZ610089 |
| YHGY433 Lipocrea fusiformis | PX230063 | Nephila pilipes YN1 | DQ779278 |
| YHGY495 Lipocrea fusiformis | PX230065 | Nephila pilipes YN2 | DQ779279 |
| YHGY507 Lipocrea fusiformis | PX230066 | Nephila pilipes YN3 | DQ779280 |
| YHGY508 Lipocrea fusiformis | PX230064 | Nephila constricta C40 | HQ441931 |
| YHGY208 Lipocrea guiyang | PX230067 | Nephila constricta NEP | KC849087 |
| YHGY209 Lipocrea guiyang | PX230068 | Paralarinia sp. 199 DD-2019 | MK420147 |
| YHGY428 Lipocrea guiyang | PX230069 | Pycnacantha fuscosa 256 | MK420160 |

**
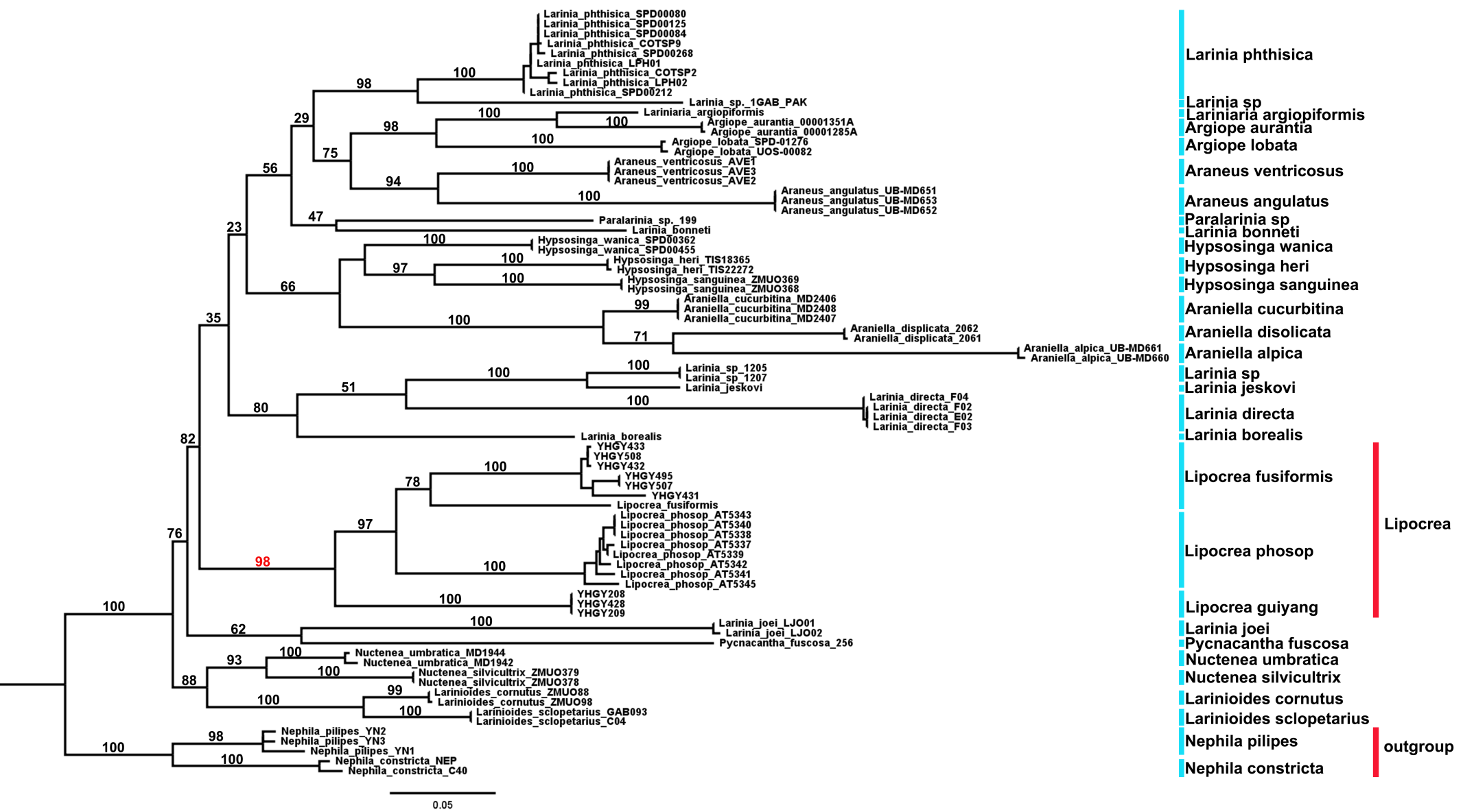
**Fig. S1. Maximum-likelihood tree constructed based on the data in Table S1. Node numbers represent bootstrap support values.
